# Preoperative MRI-radiomics features improve prediction of survival in glioblastoma patients over MGMT methylation status alone

**DOI:** 10.18632/oncotarget.26578

**Published:** 2019-01-18

**Authors:** Florent Tixier, Hyemin Um, Dalton Bermudez, Aditi Iyer, Aditya Apte, Maya S. Graham, Kathryn S. Nevel, Joseph O. Deasy, Robert J. Young, Harini Veeraraghavan

**Affiliations:** ^1^ Department of Medical Physics, Memorial Sloan Kettering Cancer Center, New York, New York, USA; ^2^ Department of Neurology, Memorial Sloan Kettering Cancer Center, New York, New York, USA; ^3^ Department of Neurology, Indiana University School of Medicine, Indianapolis, Indiana, USA; ^4^ Department of Radiology, Memorial Sloan Kettering Cancer Center, New York, New York, USA; ^5^ Brain Tumor Center, Memorial Sloan Kettering Cancer Center, New York, New York, USA

**Keywords:** magnetic resonance imaging, radiomics, glioblastoma, MGMT, survival analysis

## Abstract

**Background:**

Glioblastoma (GBM) is the most common malignant central nervous system tumor, and *MGMT* promoter hypermethylation in this tumor has been shown to be associated with better prognosis. We evaluated the capacity of radiomics features to add complementary information to *MGMT* status, to improve the ability to predict prognosis.

**Methods:**

159 patients with untreated GBM were included in this study and divided into training and independent test sets. 286 radiomics features were extracted from the magnetic resonance images acquired prior to any treatments. A least absolute shrinkage selection operator (LASSO) selection followed by Kaplan-Meier analysis was used to determine the prognostic value of radiomics features to predict overall survival (OS). The combination of *MGMT* status with radiomics was also investigated and all results were validated on the independent test set.

**Results:**

LASSO analysis identified 8 out of the 286 radiomic features to be relevant which were then used for determining association to OS. One feature (edge descriptor) remained significant on the external validation cohort after multiple testing (p=0.04) and the combination with *MGMT* identified a group of patients with the best prognosis with a survival probability of 0.61 after 43 months (p=0.0005).

**Conclusion:**

Our results suggest that combining radiomics with *MGMT* is more accurate in stratifying patients into groups of different survival risks when compared to with using these predictors in isolation. We identified two subgroups within patients who have methylated *MGMT*: one with a similar survival to unmethylated *MGMT* patients and the other with a significantly longer OS.

## INTRODUCTION

Glioblastoma (GBM) is the most common malignant central nervous system tumor (46.6%) and has a poor prognosis with only 5.5% (95% CI 5.2%-5.8%) survival at 5 years [1]. Imaging plays an important role in determining patient diagnosis, prognosis, risk assessment, response evaluation and follow up [2]. The standard treatment consists of surgical resection followed by radiotherapy with concomitant and adjuvant chemotherapy (temozolomide) [3]. Recently, several molecular biomarkers have been identified to predict prognosis and outcomes to therapy as well as to guide personalized therapies [4, 5]. The hypermethylation of the *MGMT* promoter (O^6^-methylguanine–DNA methyltransferase), which results in the silencing of this DNA repair enzyme, provides key prognostic data and has been shown to be associated with better prognosis and better response to temozolomide [6, 7]. We studied the utility of combining pre-treatment, diagnostic magnetic resonance imaging (MRI)-based radiomics features with *MGMT* methylation to further stratify patient prognosis.

Previous studies have applied qualitative visual feature descriptors assigned by radiologists from MRI scans, such as those based on the VASARI (Visually AcesSAble Rembrandt Images) criteria, showing that these may predict patient outcomes [8, 9]. Recently, radiomics features of MR images have been used to extract quantitative measures of whole tumor appearance heterogeneity to predict outcomes in multiple cancers including GBM [10]. Radiomics involves the extraction of quantitative feature descriptors of tumor appearance through edges, textures, shape and histogram features through automated high-throughput computer analysis of medical images and has been applied to predict outcomes in several cancers [10–12]. In brain cancer, radiomic features have shown feasibility to predict outcomes including survival [13–18].

Radiomics when applied to predict underlying genotypic features is called radiogenomics [19–22] and has been used to non-invasively detect association of images with underlying molecular markers such as *MGMT* methylation, *IDH1* mutation and 1p/19q co-deletion [13, 16, 18, 23, 24]. In our study, we evaluated whether the combination of the molecular biomarker *MGMT* and image-based tumor heterogeneity computed using radiomics analysis would produce more accurate overall survival (OS) classification than *MGMT* alone. We focused on the capacity of radiomics to improve the effectiveness of *MGMT* methylation as a biomarker to identify patient groups with distinct survivals among the patients with or without *MGMT* methylation. Our aim was to build an easy-to-compute and interpretable model incorporating the most relevant radiomics features to augment the capacity of *MGMT* methylation as biomarker of outcome.

**Figure 1:**
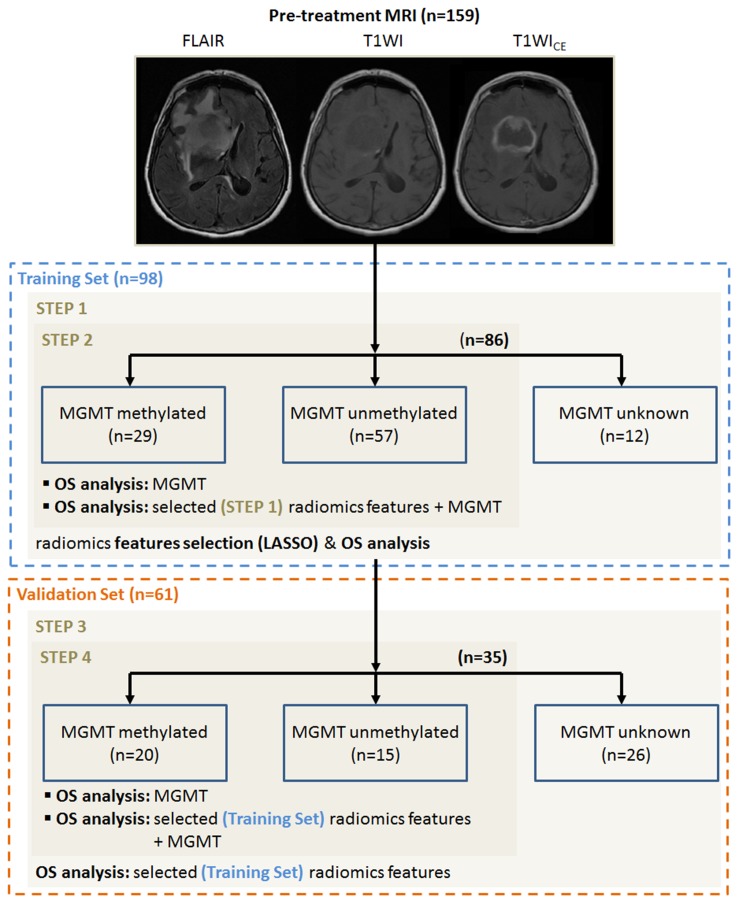
Workflow of the study divided on four steps Training cohort: 1) Radiomics feature selection using the Least Absolute Shrinkage and Selection Operator (LASSO) regression model and prognosis value of these features for the OS prediction 2) Prognosis value of MGMT and combination of MGMT and radiomics features for the OS prediction. Validation cohort: 3) Validation of the prognosis value of the selected radiomics features for the OS prediction. 4) Prognosis value of MGMT and the combination of MGMT and radiomics features for the OS prediction on the validation cohort.

Analysis consisted of several steps including relevant features selection followed by a univariate analysis to construct models that could potentially be incorporated into routine clinical workflows to inform patient prognosis and guide treatment decision making. All the analyses were performed on a training cohort and then validated on an independent validation cohort following the protocol described in Figure 1.

## RESULTS

### Patient characteristics

Median patient age at diagnosis was 61.5 years (range, 18-87) and 40% were female. Seventy percent of the patients had a KPS score >80%. Among the 121 patients with known *MGMT* status, 40% were methylated. A large majority of the patients were *IDH* wild-type (n=137). All patient characteristics including details specific to training and external validation cohorts are summarized in Table 1.

**Table 1:** Patient characteristics and outcome

		Training Cohort (n=98)	Validation Cohort (n=61)	All (n=159)
Age (median [range])		64y [20–87]	61y [18–80]	61.5y [18–87]
**Gender**				
	F	38 (39%)	26 (43%)	64 (40%)
	M	60 (61%)	35 (57%)	95 (60%)
**KPS (%)**				
	50%	1 (1%)	1 (2%)	2 (1%)
	60%	5 (5%)	7 (11%)	12 (7%)
	70%	23 (23%)	2 (3%)	25 (16%)
	80%	26 (27%)	25 (41%)	51 (32%)
	90%	31 (32%)	4 (7%)	35 (22%)
	100%	12 (12%)	13 (21%)	25 (16%)
	Unknown	0 (0%)	9 (15%)	9 (6%)
**MGMT status**				
	Unmethylated	57 (58%)	15 (25%)	73 (46%)
	Methylated	29 (30%)	20 (33%)	49 (31%)
	Unknown	12 (12%)	26 (42%)	37 (23%)
**IDH status**				
	Wild-type	90 (92%)	47 (77%)	137 (86%)
	Mutant	5 (5%)	3 (5%)	8 (5%)
	Unknown	3 (3%)	11 (18%)	14 (9%)
**Extent of resection**				
	Biopsy	0 (0%)	10 (16%)	10 (6%)
	Subtotal resection	52 (53%)		
	Near total resection	16 (16%)	51 (84%)	149 (94%)
	Gross total resection	30 (31%)		
**OS**				
	median [range]	587d [79 - 1324]	478d [6 - 2368]	567d [6 - 2368]
	1yr survival probability	71%	63%	68%
	2yr survival probability	43%	25%	36%
	3yr survival probability	28%	17%	26%
**Vital Status**				
	Dead	56 (57%)	45 (74%)	101 (64%)

### Feature selection

**Figure 2:**
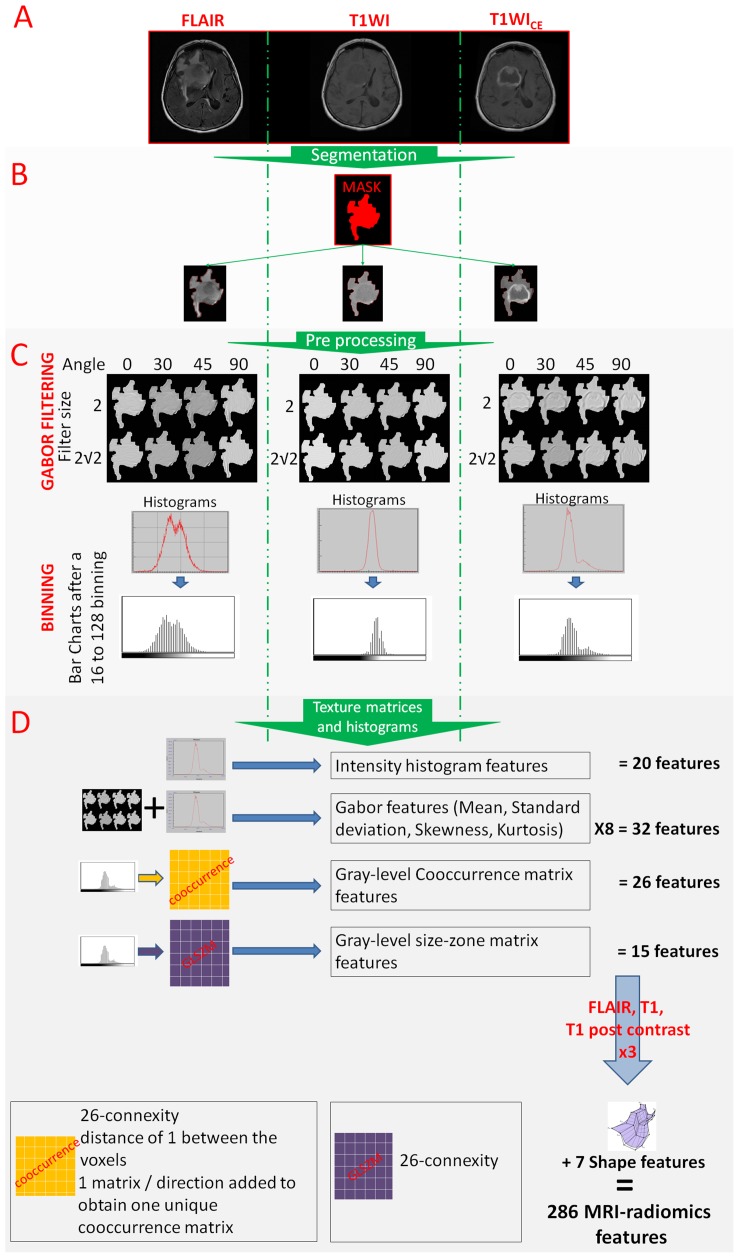
From the image acquisition to the radiomics features extraction **(A)**. Acquisition of T1WI, T1WI_CE_ and FLAIR sequences. **(B)**. Image segmentation using simultaneously the information from the 3 sequences (in red) and reporting this segmentation on all sequences. **(C)**. Image post treatments: Gabor filtering and binning. **(D)**. Radiomics feature extraction.

286 MRI-radiomics features (Figure 2D) were used in a least absolute shrinkage selection operator (LASSO) regression model to reduce the number of features in the training cohort to identify the features most relevant to outcome. Different bin sizes of MRI intensity were found to have a limited impact on feature selection: the model using a 128-bin setting selected the same set of features as the model using a 32 or 64-bin setting, and there was also a large overlap in the set of selected features with that of the model using a 16-bin setting (Supplementary Table 1).

The model based on a 128-bin setting selected 8 MRI-radiomic features as relevant to outcome: 1 feature from fluid attenuation inversion recovery (FLAIR) images (intensity histogram feature category: interquartile range), 2 features from T1-weighted images (T1WI) images (Gray-Level Co-occurrence Matrix (GLCM) feature category: cluster prominence and difference variance), 2 features from the contrast-enhanced T1-weighted (T1WI_CE_)(Gabor feature category: G_θ=0, f=2_ Skewness and G_θ=0, f=2_ Mean) and 1 feature from the T1WI_CE_ images (intensity histogram feature category: 10^th^ percentile), and 2 shape features (sphericity and surface-to-volume ratio).

### Overall survival analysis in training cohort

#### *MGMT* methylation status

**Figure 3:**
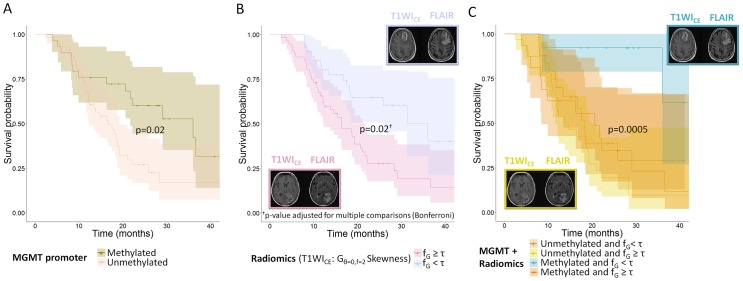
Overall survival curves for **(A)** Methylation of the MGMT promoter (n=86 patients); **(B)** Edge enhancement the tumor in T1WI_CE_ images (skewness of histogram after Gabor filtering with a direction of 0° and a frequency of 2 – patients are split according to the optimal cut-off value τ = −0. 49), (n=98 patients); **(C)** Combination of the methylation status of the MGMT promoter and edge enhancement in T1WI_CE_ images (skewness of histogram after Gabor filtering with a direction of 0° and a frequency of 2, the reported p-value is for the difference in prognosis for the patients with methylation and heterogeneity under the median vs. the rest, (n=86 patients).

MGMT methylation alone was associated with improved OS with a median survival of 36.0 months for methylated patients and 16.8 months for unmethylated patients (p=0.02) (see Figure 3A).

#### Radiomic features

Table 2 presents the optimal thresholds for each selected feature obtained with Receiver Operating Characteristic (ROC) curve analysis. Of the 8 selected features, 4 were significantly associated with OS after adjusting for multiple comparisons using the Bonferroni procedure: 2 shape features (sphericity and surface-to-volume ratio) and 2 features from T1WI_CE_ images (G_θ=0, f=2_ Skewness and G_θ=0, f=2_ Mean). Tumors with high sphericity (> 0.73) or surface-to-volume ratio < 1.73 were associated with a longer median survival (36.0 months) vs. tumors with low sphericity (17.5 months) and tumors with large surface-to-volume ratio (15.5 months), respectively. Patients with the highest edge enhancement on T1WI_CE_ (i.e., G_θ=0, f=2_ Skewness value lower than the optimal threshold [−0.49]) had a longer median survival (36.0 months) vs. their counterpart (16.3 months) (Figure 3B). Median survival times are reported in Table 2.

**Table 2:** Overall survival analysis in the training and validation sets. The 8 features selected by the LASSO regression model were used in the training set and the 4 significant features in the training set were used in the validation set. All p-values were corrected for multi-testing by the Bonferroni procedure

				Testing set				Validation set			
				median survivals (95% LCL; 65% UCL) (months)				median survivals (95% LCL; 65% UCL) (months)			
Image sequence	Feature category	Feature name	cut-off value	group 1 (≥ cut-off)	group 2 (<cut-off)	p-values^†^	group sizes	group 1 (≥ cut-off)	group 2 (< cut-off)	p-values^†^	group sizes
FLAIR	Intensity histogram	Interquartile range	175.5	21.7 (18.4; NA^‡^)	16.3 (12.1; 29.0)	0.8	52/46	-	-	-	-
T1WI	GLCM	Cluster prominence	1.77E+07	25.9 (21.0; NA^‡^)	17.5 (14.6; 28.3)	0.32	35/63	-	-	-	-
		Difference variance	253.7	NA^‡^ (11.6; NA^‡^)	18.7 (16.3; 25.9)	0.24	22/76	-	-	-	-
T1WI_CE_	Intensity histogram	10th percentile	29.5	18.2 (15.5; 22.2)	NA^‡^ (NA^‡^; NA^‡^)	0.06	72/26	-	-	-	-
	Gabor features	G_θ=0, f=2_ Mean	144.1	29.0 (18.4; NA^‡^)	16.8 (12.1; 22.2)	0.02	54/44	15.7 (13.3; 22.3)	14.1 (6.8; NA^‡^)	1	51/10
		G_θ=0, f=2_ Skewness	-0.49	16.3 (12.4; 21.7)	36.0 (25.9; NA^‡^)	0.02	56/42	12.2 (8.5; 19.7)	20.6 (15.7; NA^‡^)	0.04	31/30
NA	Shape	Sphericity	0.73	36.0 (19.3; NA^‡^)	17.5 (12.4; 22.1)	0.007	47/51	16.0 (13.9; 22.3)	8.5 (6.8; NA^‡^)	0.8	50/11
		Surface to volume ratio	1.73	15.5 (11.6; 21.0)	36.0 (19.3; NA^‡^)	0.002	34/64	20.6 (13.3; NA^‡^)	14.7 (11.7; 20.8)	1	16/45

^†^Significance of difference in the OS between the two groups (p-values were corrected for multiple comparisons with Bonferroni procedure)

^‡^NA values appears when the survivorship function does not reach 0.5

95% LCL and 95% UCL are the 95% lower and upper confidence limits around the median survivals

#### Combination of MGMT and Radiomics

The combination of *MGMT* methylation status and the selected G_θ=0, f=2_ Skewness radiomics feature identified a subgroup of patients with a better prognosis than their counterparts. Patients with *MGMT* methylation and high negative skewness of Gabor edge enhancement had a better prognosis (survival probability of 0.61 after 43 months of observation) compared with other patients (survival probability of 0.15 and median survival of 17.5 months, (95% CI: 13.4, 21.0)) (p=0.0005). On the other hand, patients with *MGMT* methylation and G_θ=0, f=2_ Skewness > optimal threshold [−0.49]) did not have a significantly different prognosis than patients without *MGMT* methylation (p=0.5) (Figure 3C). *MGMT* methylation status combined with the remaining selected features (sphericity, surface-to-volume ratio and G_θ=0, f=2_ Mean) did not identify subgroups of patients with significantly different prognosis.

### Overall survival in the validation cohort

#### *MGMT* methylation status

**Figure 4:**
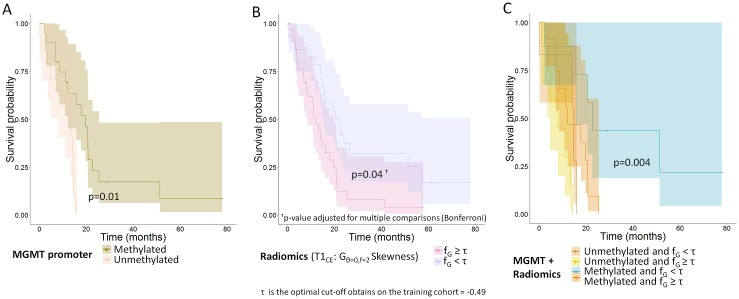
Overall survival curves for a validation cohort **(A)** Methylation of the MGMT promoter (n=35 patients); **(B)** Edge enhancement in T1WI_CE_ images (skewness of histogram after Gabor filtering with a direction of 0° and a frequency of 2). Patients are split using the optimal cut-off value of −0.49 obtained from the training cohort (n=61 patients); **(C)** Combination of the methylation status of the MGMT promoter and edge enhancement in T1WI_CE_ images (skewness of histogram after Gabor filtering with a direction of 0° and a frequency of 2, the reported p-value is for the difference in prognosis for the patients with methylation and heterogeneity under the median vs. the rest, (n=35 patients).

In the validation cohort, *MGMT* methylation alone remained a significant prognostic factor with a median survival of 19.7 months for methylated patients vs. 13.3 months for unmethylated patients, p=0.01 (Figure 4A).

#### Radiomic features

Using the optimal thresholds identified in the training dataset, only the G_θ=0, f=2_ Skewness feature from T1WI_CE_ images remained significantly associated with OS after adjusting for multiple comparisons with the Bonferroni procedure (Table 2). Similar to the training set, patients with highest edge enhancement on T1WI_CE_ (G_θ=0, f=2_ skewness< optimal threshold [−0.49], p=0.04) had a longer median survival of 20.6 months compared with 12.2 months for the group with low edge enhancement (Figure 4B).

#### Combination of MGMT and G_θ=0, f=2_ skewness

The validation set confirmed the results of the training set: those patients who had both *MGMT* methylation and high negative skewness of Gabor edge enhancement had a longer median OS of 22.7 months vs. patients who did not and who had a median OS of 12.2 months (p=0.004) (Figure 4C).

## DISCUSSION

Our study differed from the typical radiogenomic studies which aim to find correlations between radiomics features with genotypic expressions [16, 18, 23, 24]. Instead, we examined the synergistic value of adding radiomics to *MGMT* methylation status to predict prognosis. Increasingly, studies have employed next generation sequencing to show the predictive utility of underlying tumor genotypic and transcriptomic profiles for predicting survival [25, 26]. Next generation sequencing studies are not readily available at all centers, however, and may be limited by the available amount of tumor tissue. In contrast, *MGMT* status is widely available via PCR based methods. We therefore tested our hypothesis that textural intra-tumor heterogeneity, tumor shape and intensity histogram features computed from radiomics analysis could be used to augment efficacy of predictive biomarkers such as *MGMT* status.

Results from our study showed that Gabor features extracted from T1WI_CE_ MRI complements the findings using qualitative visual measures using the VASARI criteria [27]. For instance, the length of the lesion's major axis on FLAIR, the proportion of contrast-enhanced on T1WI_CE_ and ring enhancement assessed from T1WI_CE_ images have shown to be associated with survival [28] and/or *MGMT* promoter methylation in GBM [29].

We developed a model combining radiomics and *MGMT* methylation status to predict OS and showed that the combined model predicted survival in an independent validation cohort using the median feature values extracted from the training cohort. Our study identified the skewness of the Gabor filtered edge image (G_θ=0, f=2_ Skewness) computed from the T1WI_CE_ as a significant predictor of survival. High negative skewness of Gabor edges was associated with longer OS. Gabor edges are directional sensitive edge detectors and large negative skewness indicates that patients with longer OS exhibited a more homogeneous distribution of edges in their tumors compared with those with shorter OS (see Supplementary Figure 1).

In contrast to prior works using radiomics analysis which showed the prognostic value of measures including GLCM [30] and GLSZM (Gray-Level Size-Zone Matrix) features for OS prediction [31], none of these measures were found to be relevant in our study, even if two were selected by the LASSO regression model. We believe that these features were not selected or not found to have a significant prognostic value due to large variations in MR images, such as the voxel size or the magnetic strength, within our cohort and within the multi-institutional cohort. Indeed, it is possible that such effects influenced the value of these features more than patients’ prognosis.

Second, tumor segmentation was performed by simultaneously including all three sequences in order to extract the entire tumor extent including any visible edema on the FLAIR sequence. The Gabor edges were better than the whole tumor summary measures such as the GLCM features to quantify the appearance difference between tumor bulk and edema.

The orientation (0 degree) of the Gabor filter that was selected by our model may be explained with the shape of the tumors that have their longest diameter in the back to front orientation, probably because of the shape of the brain. In addition, the results for this feature were confirmed on the validation cohort using the median value found on the training cohort as the threshold.

The shape features (sphericity and surface-to-volume ratio) were not found to be significantly associated with OS in the validation cohort. This result might be explained by the imbalance in cohort size within the group after splitting the training set by the optimal threshold. Further investigations are needed to evaluate the prognostic value of shape descriptors from MRI images in GBMs.

However, the selection of eight features among the 286 investigated features does not mean that these selected features are the only relevant features. Indeed, LASSO selects and ranks independent features in the order of their relevance for the predicting the outcome variable. In other words, features that are correlated with the top-ranking features are removed, which reduces the false discovery due to multi-testing.

Our analysis identified OS-relevant radiomics features. However, unlike other studies that have reported a significant correlation between *MGMT* status and radiomics features [23, 32], we found no such correlation. Because *MGMT* status and OS are indirectly connected, especially for the survival during the first months [7], it is possible that features correlated with *MGMT* status were not selected in our model.

The combination of the G_θ=0, f=2_ Skewness radiomic feature and *MGMT* status identified a group of patients with a better prognosis in both the training and validation cohorts. These patients had methylated *MGMT* and a high negative value of Gabor edge skewness. At the opposite, methylated *MGMT* patients without a high value of Gabor edge skewness had OS indistinguishable from unmethylated *MGMT* patients. We believe that the radiomic feature quantifies the sharpness of the tumor boundary on the T1WI_CE_ and might reflect the degree of tumor infiltration. An interesting extension of this study will be to focus on the correlation between altered genes or pathways and the measurements of tumor edges.

Our radiomics approach differs from others in a few aspects. First, we used automated tumor segmentations with machine learning that was then edited or approved with minimal user input. Second, the tumor segmentations and ultimately the radiomics features were calculated from a single segmented volume combining T1WI, T1WI_CE_ and FLAIR. Such a segmented volume in fact includes the edema visible on FLAIR images that will be excluded when using only T1WI, and T1WI_CE_ scans. Inclusion of edema components helps the features to quantify the strength of tumor edges.

Our study suffers from some limitations. First, features were extracted without histogram standardization techniques and voxel resampling techniques which could have resulted in the decrease or the loss of the prognostic value of several features [33, 34]; however, this was done because our goal was to build models that could be easily used in a clinical practice across institutions and which would be applicable for new patients without constraints in the image acquisition protocol. Second, we employed one segmentation for all sequences. Extracting features from different segmentations derived from different sequences could have potentially resulted in capturing different aspects of the tumor microenvironment. We plan to pursue such approach especially for correlating image-based features to the genomic pathways in the future. Third, we investigated only one feature selection technique (LASSO) with the dead or alive status of the patients as a binomial outcome. This technique allowed us to identify features that are of potential interest, but it cannot guarantee the best feature selection. However, this method was chosen based on the work of Deist et al., who reported that random forest and elastic net logistic regression yielded higher discriminative performance compared with several other machine learning methods [35]. Fourth, although our cohort consisted mostly of *IDH* wildtype GBMs, we included a few *IDH* mutant GBMs that represented 5% of our patients and is concordant with the reported proportion of this rare mutation in primary GBMs [36]. Nevertheless, the model that we developed for untreated GBMs was able to successfully predict survival regardless of *IDH* status. Supplementary Table 2 provides the repartition of *IDH* mutant patients into the considered groups and no bias due to these patients was found.

Our study showed the combined value of radiomics with *MGMT* in predicting OS in patients diagnosed with GBM from pre-treatment MRI using cross-validation training and independent test sets. Our results highlight that radiomics features possibly contain information that is not supplied by molecular markers such as *MGMT* methylation alone. Particularly, we identified that the methylated *MGMT* patients can be split into two groups with significantly different survival times. Future studies will focus on methylated *MGMT* glioblastoma patients and the ability for the radiologist to visually determine analogs to the radiomic characteristics that we have identified.

## MATERIALS AND METHODS

### Patients

A total of 159 patients with untreated GBM and pre-treatment MRI scans were retrospectively analyzed in our study, from Memorial Sloan Kettering Cancer Center (MSK=112) and from The Cancer Imaging Archive (TCIA=47). All patients were scanned with MRI consisting of T1WI, T1WI_CE_, and FLAIR images. Ninety-eight patients from MSK were included in the training set for our study; 78 underwent surgery followed by concomitant and adjuvant radiochemotherapy (30 fractions of 6000cGy and temozolomide) according to the standard Stupp protocol [3] and the remaining 20 others patients were treated with hypofractionated radiation (15 fractions of 4150±610cGy), Optune tumor treating fields (Novocure, St. Helier, Jersey) and/or bevacizumab (Genetech, San Francisco, CA). After determining the training cohort for our study, 61 patients (14 MSK + 47 TCIA) with brain MRIs and unknown treatments (for the TCIA) were used to build an independent validation cohort. The analyzed outcome was overall survival (OS) defined as defined as the time between the date of diagnosis and the last follow-up date or the date of event (e.g., death). Radiomics analysis was performed in both the training and validation cohorts. To determine if the combined performance of radiomics and *MGMT* status would better predict prognosis, we used a reduced cohort of 121 patients (86 for training and 35 for validation) with known MGMT status (see Table 1).

#### Memorial Sloan Kettering Cancer Center Patients

This retrospective study was approved by the MSK institutional review board with a waiver of written informed consent. MR images were acquired using Discovery MR750w and MR450w scanners (GE Medical Systems, Milwaukee, WI) with a standard head or neurovascular coil. The image acquisition details for this cohort are provided in Supplementary Table 3. All patients underwent maximal safe resection as per standard of care. Tumor DNA was tested using a polymerase chain reaction (PCR) based assay for *MGMT* promoter hypermethylation in region +28 to +47 from the translation start site in exon 1.

#### TCIA Patients

From the GBM patients in the TCIA database (n=262), we excluded those patients who (a) were missing at least one of the three sequences, (b) had images with severe motion artifacts, and (c) were either recurrent GBMs or determined to be post-operative MRIs. This resulted in a total of 47 patients between 1998 to 2011 with pre-operative MRIs consisting of T1WI, T1WI_CE_ and FLAIR images (included patient IDs are in the supplementary material). The image acquisition details for this cohort are provided in Supplementary Table 1. *MGMT* methylation status of these patients was obtained from the TCGA database using the corresponding patient IDs. Further details of the analyzed patients from TCIA data can be downloaded on the TCIA website: https://wiki.cancerimagingarchive.net.

### Radiomics features extraction

All tumors were segmented using grow-cut segmentation combined with active machine learning [37]. The segmentations were validated by an experienced neuroradiologist with >18 years of experience with MRI and brain tumor imaging (R.J.Y) and manually adjusted where required prior to radiomics feature extraction (Figure 2B).

#### Tumor segmentation

The algorithm from Veeraraghavan and Miller 2011 [37] was modified to simultaneously process multiple MR image sequences consisting of T1WI, T1WI_CE_ and FLAIR images. Prior to segmentation, the image sequences were co-registered with a rigid registration using our in-house implementation using a C++ wrapper function implemented around the Insight ToolKit [38].

Next, starting from user-drawn brush strokes to identify the tumor and background regions, the algorithm generated segmentation of the tumor by automatically combining image intensities from three sequences consisting of FLAIR, T1w pre and T1w post-contrast images. To generate the segmentation, the algorithm learned a model of the tumor through brush stroke inputs using a support vector machine (SVM) algorithm, following which any regions that were difficult to segment were automatically identified by the algorithm and presented as queries to the user to indicate whether those regions corresponded to tumor or background. Upon user input in some of those regions, the algorithm automatically refined the tumor segmentation.

#### Radiomic features

Radiomic features were extracted using the CERR software [39], which was demonstrated to be compliant with the Image Biomarkers Standardization Initiative (IBSI) nomenclature [40]. The MR intensities in all images were scaled to Q=128 values using:

$$V_{Q}\left(x\right)=round\left(\frac{\left(Q-1\right).\left(x-V_{min}\right)}{V_{max}-V_{min}}+1\right)$$

With V_min_ and V_max_ the minimum and maximum intensity value in the tumor segmentation (Figure 2C). Additionally, Gabor filtering was applied to extract edge maps from the images using four orientations (θ=0°, 30°, 45° and 90°) and with a frequency f of 2 and 2√2 [41]. Gabor filtering consists of applying bandpass filtering operation to extract edges in the image. The Gabor edges are directional sensitive filters and are composed of a Gaussian envelope function superimposed on a sinusoidal wave. In two dimensions, Gabor filters can be used to determine the edge response along various orientation (or angles) and scales (or bandwidths) of the Gaussian function. Using four orientations and two bandwidths on the three different MRI sequences resulted in twenty-four Gabor edge images.

A total of 286 MRI-radiomics texture, shape, edge and intensity histogram features were extracted from the unscaled, scaled and Gabor edge filtered MRIs from all three sequences (Figure 2D). The full list of extracted features is provided in the supplementary file. First-order features consisted of intensity histogram features (n=20) computed from the unscaled MRIs. The scaled MRI images were used to compute GLCM [42] and GLSZM [43]. An offset of one voxel in all directions and 26-connexity was used to extract the co-occurrence matrices. One matrix was built to describe the entire tumor volume using 3D neighbors and one unique matrix for all directions (using the same merging method as described in the IBSI [40]), and then used to extract GLCM features [42]. Similarly, one GLSZM was produced and then used for extracting tumor heterogeneity features [43]. In addition, four main histogram metrics (mean, standard deviation, skewness and kurtosis) were extracted from the Gabor edge maps.

### Statistical analysis

Our study was designed in accordance with the guidelines proposed by Vallières et al. for responsible radiomics research for faster clinical translation [44]: we focused particularly 1) to describe precisely the workflow from the image acquisition to the feature extraction and 2) to design a study with an independent validation and with corrections for multiple testing comparison.

All statistical analyses were performed using R software (v3.4.3) and the glmnet, roc and survival packages. Feature selection was performed implicitly using the Least Absolute Shrinkage and Selection Operator (LASSO: elastic net logistic regression with the mixing parameter equal to 1) [45] regression model with the patient's dead or alive (56% vs 42% of patients) status as a binomial response variable and a 10-fold cross-validation was used for the tuning parameter (λ). Prior to fitting the model, the features were transformed to be in the same scale using the standardization method integrated in the glmnet package. The features found to be relevant by the model were those that were assigned non-zero weights by the model. Selected features were then used to compute their association with OS. Kaplan-Meier analysis was used to determine the association between the radiomics features and OS by dichotomizing the patients using the best threshold (Youden index) obtained from ROC curves analysis of the individual features. The p-values were corrected for multiple comparisons using the Bonferroni method for controlling the false discovery rate. Only p-values <0.05 after adjustment for multiple comparisons were considered significant. We also combined the relevant radiomics features identified using the LASSO method with the *MGMT* molecular biomarker to determine whether the combined model produced better discrimination between patients by overall survival.

An independent validation set was used to validate the results on radiomics and on the combination of radiomics with *MGMT* for OS prediction. In this cohort, the previously selected radiomics features were dichotomized using the median value reported in the discovery set.

## SUPPLEMENTARY MATERIALS FIGURES AND TABLES



## Abbreviations

FLAIR: Fluid Attenuation Inversion Recovery
GBM: Glioblastoma
GLCM: Gray-Level Co-occurrence Matrix
GLSZM: Gray-Level Size-Zone Matrix
*IDH*: isocitrate dehydrogenase
LASSO: Least Absolute Shrinkage and Selection Operator
*MGMT*: O^6^-methylguanine–DNA methyltransferase
MRI: Magnetic Resonance Imaging
MSK: Memorial Sloan Kettering
PCR: Polymerase Chain Reaction
PFS: Progression-free Survival
T1WI, T1WI_CE_: T1-weighted images
TCGA/TCIA: The Cancer Genome Archive/The Cancer Imaging Archive
OS: Overall Survival
ROC: Receiver Operating Characteristic
SVM: Support Vector Machine
VASARI: Visually AcesSAble Rembrandt Images

## References

[1] Ostrom QT, Gittleman H, Xu J, Kromer C, Wolinsky Y, Kruchko C, Barnholtz-Sloan JS (2016). CBTRUS Statistical Report: Primary Brain and Other Central Nervous System Tumors Diagnosed in the United States in 2009–2013. Neuro Oncol.

[2] Weller M, van den Bent M, Tonn JC, Stupp R, Preusser M, Cohen-Jonathan-Moyal E, Henriksson R, Rhun EL, Balana C, Chinot O, Bendszus M, Reijneveld JC, Dhermain F (2017). European Association for Neuro-Oncology (EANO) guideline on the diagnosis and treatment of adult astrocytic and oligodendroglial gliomas. Lancet Oncol.

[3] Stupp R, Mason WP, van den Bent MJ, Weller M, Fisher B, Taphoorn MJ, Belanger K, Brandes AA, Marosi C, Bogdahn U, Curschmann J, Janzer RC, Ludwin SK (2005). Radiotherapy plus Concomitant and Adjuvant Temozolomide for Glioblastoma. N Engl J Med.

[4] Gonzalez de Castro D, Clarke PA, Al-Lazikani B, Workman P (2013). Personalized Cancer Medicine: Molecular Diagnostics, Predictive biomarkers, and Drug Resistance. Clin Pharmacol Ther.

[5] Weller M, Stupp R, Reifenberger G, Brandes AA, van den Bent MJ, Wick W, Hegi ME (2010). MGMT promoter methylation in malignant gliomas: ready for personalized medicine?. Nat Rev Neurol.

[6] Stupp R, Hegi ME, Mason WP, van den Bent MJ, Taphoorn MJ, Janzer RC, Ludwin SK, Allgeier A, Fisher B, Belanger K, Hau P, Brandes AA, Gijtenbeek J (2009). Effects of radiotherapy with concomitant and adjuvant temozolomide versus radiotherapy alone on survival in glioblastoma in a randomised phase III study: 5-year analysis of the EORTC-NCIC trial. Lancet Oncol.

[7] Hegi ME, Diserens AC, Gorlia T, Hamou MF, de Tribolet N, Weller M, Kros JM, Hainfellner JA, Mason W, Mariani L, Bromberg JEC, Hau P, Mirimanoff RO (2005). MGMT Gene Silencing and Benefit from Temozolomide in Glioblastoma. N Engl J Med.

[8] Nicolasjilwan M, Hu Y, Yan C, Meerzaman D, Holder CA, Gutman D, Jain R, Colen R, Rubin DL, Zinn PO, Hwang SN, Raghavan P, Hammoud DA (2015). Addition of MR imaging features and genetic biomarkers strengthens glioblastoma survival prediction in TCGA patients. J Neuroradiol.

[9] Mazurowski MA, Desjardins A, Malof JM (2013). Imaging descriptors improve the predictive power of survival models for glioblastoma patients. Neuro Oncol.

[10] Lambin P, Rios-Velazquez E, Leijenaar R, Carvalho S, van Stiphout RG, Granton P, Zegers CM, Gillies R, Boellard R, Dekker A, Aerts HJ (2012). Radiomics: Extracting more information from medical images using advanced feature analysis. Eur J Cancer.

[11] Gillies RJ, Kinahan PE, Hricak H (2015). Radiomics: Images Are More than Pictures, They Are Data. Radiology.

[12] Lambin P, Leijenaar RTH, Deist TM, Peerlings J, de Jong EEC, van Timmeren J, Sanduleanu S, Larue RTHM, Even AJG, Jochems A, van Wijk Y, Woodruff H, van Soest J (2017). Radiomics: the bridge between medical imaging and personalized medicine. Nat Rev Clin Oncol.

[13] Zhou H, Vallières M, Bai HX, Su C, Tang H, Oldridge D, Zhang Z, Xiao B, Liao W, Tao Y, Zhou J, Zhang P, Yang L (2017). MRI features predict survival and molecular markers in diffuse lower-grade gliomas. Neuro Oncol.

[14] Lao J, Chen Y, Li ZC, Li Q, Zhang J, Liu J, Zhai G (2017). A Deep Learning-Based Radiomics Model for Prediction of Survival in Glioblastoma Multiforme. Sci Rep.

[15] Prasanna P, Patel J, Partovi S, Madabhushi A, Tiwari P (2017). Radiomic features from the peritumoral brain parenchyma on treatment-naïve multi-parametric MR imaging predict long versus short-term survival in glioblastoma multiforme: Preliminary findings. Eur Radiol.

[16] Lee J, Narang S, Martinez JJ, Rao G, Rao A (2015). Associating spatial diversity features of radiologically defined tumor habitats with epidermal growth factor receptor driver status and 12-month survival in glioblastoma: methods and preliminary investigation. J Med Imaging (Bellingham).

[17] Upadhaya T, Morvan Y, Stindel E, Le Reste PJ, Hatt M (2015). A framework for multimodal imaging-based prognostic model building: Preliminary study on multimodal MRI in Glioblastoma Multiforme. IRBM.

[18] Abrol S, Kotrotsou A, Salem A, Zinn PO, Colen RR (2017). Radiomic Phenotyping in Brain Cancer to Unravel Hidden Information in Medical Images. Top Magn Reson Imaging.

[19] Aerts HJ, Velazquez ER, Leijenaar RT, Parmar C, Grossmann P, Carvalho S, Bussink J, Monshouwer R, Haibe-Kains B, Rietveld D, Hoebers F, Rietbergen MM, Leemans CR (2014). Decoding tumour phenotype by noninvasive imaging using a quantitative radiomics approach. Nat Commun.

[20] Segal E, Sirlin CB, Ooi C, Adler AS, Gollub J, Chen X, Chan BK, Matcuk GR, Barry CT, Chang HY, Kuo MD (2007). Decoding global gene expression programs in liver cancer by noninvasive imaging. Nat Biotechnol.

[21] Tixier F, Cheze-Le-Rest C, Hatt M, Dufour X, Valette G, Potard G, Corcos L, Visvikis D (2015). TU-CD-BRB-10: 18F-FDG PET Image-Derived Tumor Features Highlight Altered Pathways Identified by Trancriptomic Analysis in Head and Neck Cancer. Med Phys.

[22] Gevaert O, Mitchell LA, Achrol AS, Xu J, Echegaray S, Steinberg GK, Cheshier SH, Napel S, Zaharchuk G, Plevritis SK (2014). Glioblastoma Multiforme: Exploratory Radiogenomic Analysis by Using Quantitative Image Features. Radiology.

[23] Xi Y, Guo F, Xu Z, Li C, Wei W, Tian P, Liu T, Liu L, Chen G, Ye J, Cheng G, Cui L, Zhang H (2018). Radiomics signature: A potential biomarker for the prediction of MGMT promoter methylation in glioblastoma. J Magn Reson Imaging.

[24] Korfiatis P, Kline TL, Coufalova L, Lachance DH, Parney IF, Carter RE, Buckner JC, Erickson BJ (2016). MRI texture features as biomarkers to predict MGMT methylation status in glioblastomas. Med Phys.

[25] Gonzalez-Angulo AM, Hennessy BTJ, Mills GB (2010). Future of Personalized Medicine in Oncology: A Systems Biology Approach. J Clin Oncol.

[26] Ghasemi M, Nabipour I, Omrani A, Alipour Z, Assadi M (2016). Precision medicine and molecular imaging: new targeted approaches toward cancer therapeutic and diagnosis. Am J Nucl Med Mol Imaging.

[27] VASARI- Imaging- Cancer Imaging Program - National Cancer Institute - Confluence Wiki [Internet] https://wiki.nci.nih.gov/display/cip/vasari.

[28] Gutman DA, Cooper LA, Hwang SN, Holder CA, Gao J, Aurora TD, Dunn WD, Scarpace L, Mikkelsen T, Jain R, Wintermark M, Jilwan M, Raghavan P (2013). MR Imaging Predictors of Molecular Profile and Survival: Multi-institutional Study of the TCGA Glioblastoma Data Set. Radiology.

[29] Drabycz S, Roldán G, de Robles P, Adler D, McIntyre JB, Magliocco AM, Cairncross JG, Mitchell JR (2010). An analysis of image texture, tumor location, and MGMT promoter methylation in glioblastoma using magnetic resonance imaging. NeuroImage.

[30] Chaddad A, Tanougast C (2016). Extracted magnetic resonance texture features discriminate between phenotypes and are associated with overall survival in glioblastoma multiforme patients. Med Biol Eng Comput.

[31] Li ZC, Li Q, Sun Q, Luo R, Chen Y (2017). Identifying a radiomics imaging signature for prediction of overall survival in glioblastoma multiforme. IEEE.

[32] Peeken JC, Hesse J, Haller B, Kessel KA, Nüsslin F, Combs SE (2018). Semantic imaging features predict disease progression and survival in glioblastoma multiforme patients. Strahlenther Onkol.

[33] Molina D, Pérez-Beteta J, Martínez-González A, Martino J, Velasquez C, Arana E, Pérez-García VM (2017). Lack of robustness of textural measures obtained from 3D brain tumor MRIs impose a need for standardization. PLoS One.

[34] Upadhaya T, Morvan Y, Stindel E, Le Reste PJ, Hatt M (2016). Prognosis classification in glioblastoma multiforme using multimodal MRI derived heterogeneity textural features: impact of pre-processing choices. Medical Imaging: 2016 Computer-Aided Diagnosis. International Society for Optics and Photonics.

[35] Deist TM, Dankers FJWM, Valdes G, Wijsman R, Hsu IC, Oberije C, Lustberg T, van Soest J, Hoebers F, Jochems A, Naqa IE, Wee L, Morin O (2018). Machine learning algorithms for outcome prediction in (chemo)radiotherapy: An empirical comparison of classifiers. Med Phys.

[36] Ohgaki H, Kleihues P (2013). The Definition of Primary and Secondary Glioblastoma. Clin Cancer Res.

[37] Veeraraghavan H, Miller JV (2011). Active learning guided interactions for consistent image segmentation with reduced user interactions. 2011 IEEE International Symposium on Biomedical Imaging: From Nano to Macro.

[38] Yoo TS, Ackerman MJ, Lorensen WE, Schroeder W, Chalana V, Aylward S, Metaxas D, Whitaker R (2002). Engineering and Algorithm Design for an Image Processing API: A Technical Report on ITK-the Insight Toolkit. Stud Health Technol Inform.

[39] Apte AP, Iyer A, Crispin-Ortuzar M, Pandya R, van Dijk LV, Spezi E, Thor M, Um H, Veeraraghavan H, Oh JH, Shukla-Dave A, Deasy JO (2018). Technical Note: Extension of CERR for computational radiomics: A comprehensive MATLAB platform for reproducible radiomics research. Med Phys.

[40] Zwanenburg A, Leger S, Vallières M, Löck S, Initiative for the IBS (2016). Image biomarker standardisation initiative. ArXiv161207003 Cs [Internet].

[41] Daugman JG (1985). Uncertainty relation for resolution in space, spatial frequency, and orientation optimized by two-dimensional visual cortical filters. J Opt Soc Am A.

[42] Haralick RM, Shanmugam K, Dinstein I (1973). Textural Features for Image Classification. IEEE Trans Syst Man Cybern.

[43] Thibault G, Fertil B, Navarro CL, Pereira S, Cau P, Lévy N, Sequiera J, Mari JL (2009). Texture indexes and gray level size zone matrix. Application to cell nuclei classification. https://hal.archives-ouvertes.fr/hal-01499715.

[44] Vallieres M, Zwanenburg A, Badic B, Cheze Le Rest C, Visvikis D, Hatt M (2017). Responsible Radiomics Research for Faster Clinical Translation. J Nucl Med.

[45] Tibshirani R (1996). Regression Shrinkage and Selection via the Lasso. J R Stat Soc Series B Stat Methodol.

