# Supplementary material for: Preoperative MRI-radiomics features improve prediction of survival in glioblastoma patients over MGMT methylation status alone
Source: Oncotarget. 2019 Jan 18;10(6):660–72. doi: 10.18632/oncotarget.26578 (PMC6363013; doi:10.18632/oncotarget.26578)
Supplement: Supplementary file 1 [file oncotarget-10-660-s001.pdf]

Preoperative MRI-radiomics features improve prediction of survival in glioblastoma patients over MGMT methylation status alone

SUPPLEMENTARY MATERIALS

Radiomics features

All investigates features are obtained using CERR. Preprocessing options and formula are available at this address: https://github.com/cerr/CERR/wiki

Intensity-histogram feature: minimum, maximum, mean, range, standard deviation, variance, median, skewness, kurtosis, root mean square, energy, mean absolute deviation, median absolute deviation, robust mean absolute deviation, robust median absolute deviation, 10th percentile, 90th percentile, interquartile range, coefficient of dispersion, coefficient of variation.

GLCM features: energy, entropy, contrast, homogeneity, correlation, dissimilarity, sum average, sum variance, sum entropy, cluster shade, cluster prominence, correlation (Haralick), joint entropy, joint maximum, joint average, joint variance, inverse difference, inverse difference moment, inverse variance, difference entropy, difference variance, difference average, cluster tendency, auto correlation, first measure of information correlation, second measure of information correlation.

GLSZM feature: small zone emphasis, large zone emphasis, low grey level zone emphasis, high grey level zone emphasis, small zone low grey level emphasis, small zone high grey level emphasis, large zone low grey level emphasis, large zone high grey level emphasis, grey level non-uniformity, grey level non-uniformity normalized, size-zone non-uniformity, size-zone non-uniformity, zone percentage, Grey level variance, Zone size variance

Shape feature: surface area, volume, compactness v1, compactness v2, spherical disproportion, sphericity, surface to volume ratio.

Gabor features: Mean, standard deviation, skewness, kurtosis

TCIA Patients

IDs of included patients: TCGA-02-0003, TCGA-02-0011, TCGA-02-0027, TCGA-02-0033, TCGA-02-0034, TCGA-02-0037, TCGA-02-0046, TCGA-02-0047, TCGA-02-0048, TCGA-02-0059, TCGA-02-0064, TCGA-02-0068, TCGA-02-0069, TCGA-02-0070, TCGA-02-0075, TCGA-02-0085, TCGA-02-0087, TCGA-02-0106, TCGA-06-0127, TCGA-06-0128, TCGA-06-0145, TCGA-06-0154, TCGA-06-0158, TCGA-06-0162, TCGA-06-0164, TCGA-06-0168, TCGA-06-0175, TCGA-06-0176, TCGA-06-0213, TCGA-06-6389, TCGA-08-0348, TCGA-12-0616, TCGA-12-0829, TCGA-12-1093, TCGA-12-1598, TCGA-12-3650, TCGA-14-0789, TCGA-14-1794, TCGA-14-1821, TCGA-14-1829, TCGA-14-3477, TCGA-19-0955, TCGA-19-0963, TCGA-19-1390, TCGA-19-2624, TCGA-19-2631, TCGA-19-5951.

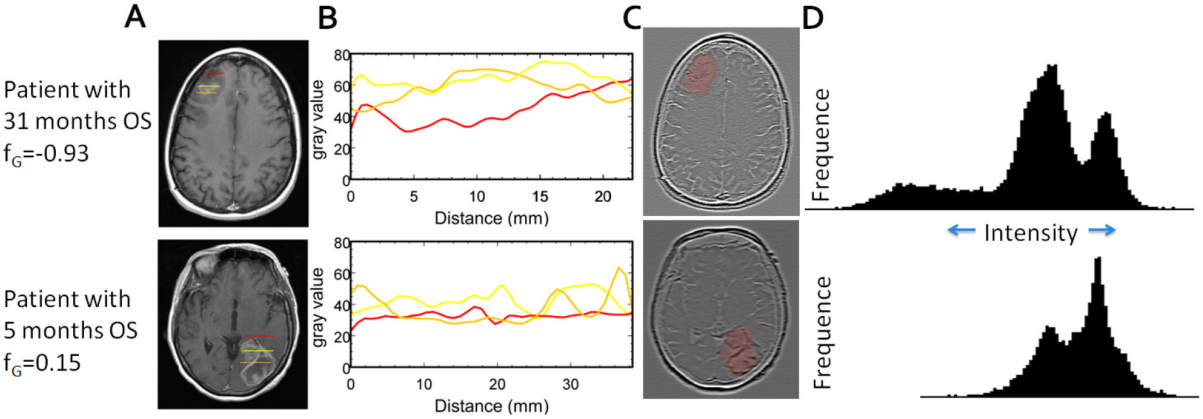

Supplementary Figure 1: Example (1) on the top - of a patient with 31 months survival and a negative skewness (-0.93) on the Gabor image ($G_{\theta=0,\ f=2}$) (2) on the bottom – of a patient with a 5 months survival and a positive skewness (0.15) on the Gabor image ($G_{\theta=0,\ f=2}$). **(A)** $T1_{CE}$ images; **(B)** intensity profile along the red, yellow and orange lines on the images in A; **(C)** Gabor images ($G_{\theta=0,\ f=2}$) **(D)** the intensity histogram distribution on the Gabor images.

Supplementary Table 1: Coefficient of each selected features by LASSO regression model with 16, 32, 64 and 128 binning

			Binning			
**Image sequence**	**Feature category**	**Feature name**	**16**	**32**	**64**	**128**
FLAIR	Intensity histogram	Interquartile range	x	-9.03E-04	-1.04E-03	-5.00E-04
	GLCM	Inverse variance	1.74E+01	x	x	x
	Gabor features	$G_{\theta=30, f=2}$ Kurtosis	x	1.36E-02	2.04E-02	x
T1WI	GLCM	Cluster prominence	-3.83E-05	-3.25E-06	-2.37E-07	-1.01E-08
		Difference variance	x	-3.42E-02	-7.86E-03	-6.59E-04
	GLSZM	Grey level non-uniformity	x	-8.39E-03	x	x
		Large zone high grey level emphasis	x	x	-3.32E-09	x
$T1WI_{CE}$	Intensity histogram	10th percentile	1.69E-02	1.28E-02	1.24E-02	1.16E-02
	Gabor features	$G_{\theta=0, f=2}$ Mean	-1.30E-02	-2.86E-02	-2.79E-02	-2.03E-02
		$G_{\theta=0, f=2}$ Skewness	1.47E-01	2.68E-01	3.15E-01	2.17E-01
NA	Shape	Spherical disproportion	1.21E-01	x	x	x
		Sphericity	-1.28E-03	-6.81E-01	-9.15E-01	-5.16E-01
		Surface to volume ratio	2.92E-01	3.95E-01	3.94E-01	3.04E-01

Supplementaty Table 2: Distribution into the 4 different groups of the 8 *IDH* averred mutant patients

Group	Number of patients
Methylated and $G_{\theta=0,\ f=2}$ Skewness <median	3
Methylated and $G_{\theta=0,\ f=2}$ Skewness >=median	3
Unmethylated and $G_{\theta=0,\ f=2}$ Skewness <median	0
Unmethylated and $G_{\theta=0,\ f=2}$ Skewness >=median	2

Supplementary Table 3: Acquisition details for the training and the independent validation patients' cohorts

		Training	Validation
Resolution (x, y)			
	[0.4-0.6]	59 (60%)	19 (31%)
	]0.6-08]	0 (0%)	11 (18%)
	]0.8-1]	39 (40%)	31 (51%)
Matrix size			
	256x256	39 (40%)	31 (51%)
	512x512	59 (60%)	15 (25%)
	others		15 (25%)
Slice thickness			
	2.5mm	0 (0%)	2 (3%)
	3mm	7 (7%)	10 (16%)
	4mm	0 (0%)	1 (2%)
	5mm	91 (93%)	48 (79%)
repetition time [mean ± std] (ms)			
	FLAIR	9147±459	9598±778
	T1WI	1479±830	669±472
	$T1WI_{CE}$	1600±851	662±435
echo time [mean ± std](ms)			
	FLAIR	135±18	135±18
	T1WI	11±6	11±3
	$T1WI_{CE}$	13±7	12±4
